# Effect of stimbiotic on growth performance, nutrient digestibility, oocyst shedding, blood profiles, and intestinal microbiota in necrotic enteritis-challenged broiler

**DOI:** 10.1080/10495398.2024.2390936

**Published:** 2024-08-16

**Authors:** Seyeon Chang, Woogi Kwak, Jihwan Lee, Seonwoong Kim, Dongcheol Song, Jaewoo An, Sehyun Park, Kyeongho Jeon, Hyuck Kim, Jinho Cho

**Affiliations:** a Department of Animal Science, Chungbuk National University, Cheongju, Republic of Korea; b Swine Science Division, National Institute of Animal Science, Rural Development Administration, Cheonan, Republic of Korea; c Department of Agricultural Economics, Chungbuk National University, Cheongju, Republic of Korea

**Keywords:** Broiler, growth performance, fecal microbiota, necrotic enteritis, stimbiotic

## Abstract

This experiment was conducted to investigate the effect of stimbiotic (STB) in broilers with necrotic enteritis (NE). A total of 180 one-day-old Arbor Acres (initial body weight of 34.81 ± 1.04 g) were used in this experiment for 32 days. All broilers were randomly allocated into six treatments, and each experimental group had 10 replicate cages with three broilers per cage. The experiment was conducted in a 2 × 3 factorial design consisting of two levels of challenge (challenge and non-challenge) and three levels of STB (0, 0.05, and 0.1%). The NE challenge significantly decreased (*P* < 0.05) growth performance, heterophil levels in blood, and intestinal lesion scores compared to the non-challenge group. Supplementation of 0.05% STB significantly decreased (*P* < 0.05) feed conversion ratio and the number of oocysts per gram of feces compared to the supplementation of 0 and 0.1% STB. At the genus level, the supplementation of 0.05% STB significantly decreased (*P* < 0.05) the abundance of *Enterobacterales* compared to the other groups on d 32. In conclusion, supplementation with 0.05% STB in a diet could positively regulate the fecal microflora and alleviate the decline in growth performance and nutrient digestibility caused by NE.

## Introduction

Enteric diseases pose a significant threat to the poultry industry, leading to production losses, heightened mortality rates, diminished welfare of birds, and an elevated risk of poultry product contamination for human consumption.^1^ Broilers mainly contract necrotic enteritis (NE) due to the gram-positive bacterium *Clostridium perfringens*.^2^^,^^3^ *C. perfringens* is a bacteria found in the intestines of healthy broilers, but it can proliferate in the intestines due to coccidiosis and unbalanced nutrients in the diet.^4^ *C. perfringens* proliferating in the large intestine moves to the small intestine and secretes toxins, causing NE.^5^ The NE typically manifests in broilers between 2 and 6 weeks of age, resulting in heightened intestinal damage and mortality rates.^6^^,^^7^ The primary approach for managing NE in poultry associated with *C. perfringens* traditionally involved the utilization of in-diet antibiotics.^8^ However, due to the development of resistance and an increase in residues due to the widespread use of antibiotics, the use of antibiotics for growth promotion in a poultry diet was banned.^9^^,^^10^ Therefore, to find new nutritional alternatives to manage NE, we focused on non-starch polysaccharides (NSP) in the diet, which is one of the causes of *C. perfringens* proliferation in the broiler intestine.^11^

Poultry diets are mainly based on grains, so they contain significant levels of NSP such as arabinoxylan.^12^ Because NSP causes reduced digestibility and intestinal microbial imbalance in broilers, the use of enzyme supplements in poultry diets can be a strategy for poultry growth.^13^^,^^14^ Xylanase (XYL) proves to be an effective addition to corn-based diets, as it effectively breaks down the β-(1-4) glycosidic bonds found in arabinoxylans.^15^ The xylo-oligosaccharides (XOS) generated by XYL function as prebiotics, directly stimulating the microbiota involved in fiber degradation and cross-feeding mechanisms.^16^ The XOS has been confirmed to be effective in improving intestinal health and intestinal microflora in broilers.^17^ Stimbiotics (STB), a mixture of XYL and XOS, are reported to be able to reduce anti-nutritional factors and partially stimulate the development of the intestinal microbiota and are emerging as a new additive.^18^ In a previous study, the supplementation of STB demonstrated the capability to diminish the inflammatory response and enhance the performance of broilers with NE challenges.^19^ However, the study on STB in broilers is still limited, and additional study is needed on the synergistic effect of mixing XYL and XOS.

Therefore, the study was conducted to assess the impact of STB on growth performance and intestinal microbiota in broilers with NE challenge. In this study, we hypothesized that (1) the deliberate induction of NE in experiments is expected to elevate the inflammatory response and diminish the performance of broilers; (2) the addition of STB as a supplement is anticipated to partially mitigate the extent of performance decline and inflammatory response; and (3) establish the most effective and economical level of STB addition.

## Materials and methods

### Ethics approval and consent to participate

The protocol for this study was reviewed and approved by the Institutioanl Animal Care and Use Committee of Chungbuk National University, Cheongju, Korea (approval no. CBNUA-2102-23-02).

### Animals, experimental design and diets

A total of 180 one-day-old Arbor Acres broilers (initial body weight [BW] of 34.81 ± 1.04 g) were obtained from a local hatchery (Cherrybro, Eumseong, Korea) and used in this experiment for 32 days. All broilers were randomly allocated into six treatments, and each experimental group had ten replicate cages with three broilers per cage. Each cage was 100 cm in width, 40 cm in depth, and 45 cm in height. The experiment initiation temperature was 33 ± 1 °C, after that, the temperature was gradually lowered to maintain 25 ± 1 °C. The experiment was conducted in a 2 × 3 factorial design of treatments consisting of two levels of challenge (challenge and non-challenge) and three levels of STB (0, 0.05, and 0.1%). All diets for the pre-starter (d 0–7), starter (d 8–14), grower (d 15–21), and finisher (d 22–32) were formulated to meet or exceed the NRC requirements (Table 1).^20^ The pre-starter phase was fed with mash feed, the starter and grower phases were fed with crumble feed, and the finisher phase was fed with pellet feed. All broilers were given ad libitum access to diet and water throughout the experiments.

**Table 1.** Ingredient composition of experimental diets.

Items	Pre-starter, d 0–7	Starter, d 8–14	Grower, d 15–21	Finisher, d 22–32
Ingredients, %				
Corn	37.6	41.6	45.2	48.9
Wheat fine	15.3	15.1	15.6	15.2
Rice pollards	2.4	2.5	2.5	2.6
Soybean meal, 45% CP	26.9	21.0	17.7	15.5
Cookie wheat flour	1.9	2.0	2.0	2.0
DDGS	5.0	7.0	6.0	5.0
Animal protein^1^	6.3	6.1	6.4	6.2
Animal fat	1.7	1.9	1.9	1.9
L-lysine	0.6	0.6	0.6	0.5
L-methionine	0.4	0.3	0.3	0.4
L-threonine	0.2	0.1	0.1	0.1
L-tryptophan	0.1	0.1	0.1	0.1
Salt	0.2	0.2	0.2	0.2
Limestone	0.5	0.6	0.5	0.5
MDCP	0.2	0.2	0.2	0.2
Liquid-Choline	0.1	0.1	0.1	0.1
Vitamin premix^2^	0.3	0.3	0.3	0.3
Mineral premix^3^	0.3	0.3	0.3	0.3
Total	100.0	100.0	100.0	100.0
Chemical composition				
AMEn, Kcal/kg	3,000	3,020	3,070	3,100
CP, %	23.3	21.3	20.2	19.1
Ether extract, %	5.5	5.9	6.0	5.8
Crude fiber, %	3.4	3.4	3.2	3.0
Crude ash, %	5.8	5.3	5.1	4.8
Calcium, %	0.9	0.8	0.8	0.7
Total phosphorus, %	0.5	0.6	0.5	0.5
Lysine, %	1.5	1.3	1.2	1.1
SAA, %	1.1	1.0	1.0	1.0

^1^Tankage meat meal, Meat-bone meal, and Poultry offal meal.

^2^Supplied per kg diet: vitamin A, 9000 IU; vitamin D_3_, 3000 IU; vitamin E, 48 mg; vitamin K, 3 mg; thiamin, 1.8 mg; riboflavin, 6 mg; pyridoxine, 3 mg; vitamin B_12_, 0.012 mg; niacin, 42 mg; folic acid, 1.2 mg; biotin, 0.24 mg; pantothenic acid, 12 mg.

^3^Supplied per kg of diet: manganese, 120 mg; zinc, 100 mg; iron, 80 mg; copper, 20 mg; iodine, 2 mg; selenium, 0.3 mg; cobalt, 0.5 mg.

DDGS: Dried distiller’s grains with soluble; MDCP: mono-dicalcium phosphate; SAA: sulfur amino acids; AMEn: nitrogen-corrected apparent metabolizable energy; CP: crude protein.

### Necrotic enteritis challenge

The experimental NE challenge model was based on a previous study.^19^ On d 14, all broilers in challenged groups were orally challenged by overdosing with coccidia and infectious bursal disease (IBD) vaccines (×10 recommended doses, respectively). Coccidia vaccine (Hipra Evalon^®^, Laboratorios Hipra, Girona, Spain) contained *Eimeria acervuline* (Strain 003, 332–450 oocysts per dose), *Eimeria brunet* (Strain 034, 213–288 oocysts per dose), *Eimeria maxima* (Strain 013, 196–265 oocysts per dose), *Eimeria necatrix* (Strain 033, 340–460 oocysts per dose) and *Eimeria tenella* (Strain 004, 276–374 oocysts per dose) strains. The freeze-dried live intermediate strain of infectious bursitis virus contained in the IBD vaccine (IBD blen^®^, Boehringer Ingelheim Animal Health USA Inc., Georgia, USA) is infectious bursal disease Winterfield 2512. In 4 d after vaccination, 3 mL of *C. perfringens* (1 × 10^7^ colony forming unit/mL) was orally challenged by dividing for 3 consecutive days (on d 18-20). The *C. perfringens* used in this experiment was *C. perfringens* type A NCTC 8798 (NCTC, National Collection of Type Cultures, London, UK). The non-challenged group broilers received the same dosage of sterile phosphate-buffered saline (PBS) via oral gavage.

### Growth performance

Broilers were weighed weekly throughout the experiment, including at the beginning of the experiment. Body weight gain (BWG) for each period was calculated. Feed intake (FI) was measured to calculate the feed conversion ratio (FCR).

### Nutrient digestibility

During the second and final weeks of the experiment, 0.2% chromium oxide (Cr_2_O_3_) was mixed as an indigestible marker in all broiler diets for analyzing nutrient digestibility. Feces and diet were collected and immediately frozen at −20 °C. At the time of analysis, fecal samples were dried at 70 °C for 48 h and then crushed through a 1 mm screen. The dry matter (DM; method 930.15) and crude protein (CP; method 984.13) of both feces and diet samples were analyzed following AOAC methods.^21^ The gross energy (GE) content was analyzed by using an adiabatic oxygen bomb calorimeter (Parr 6400 Bomb Calorimeter, Parr Instrument Co., Moline, IL, USA). Chromium levels were assessed using ultraviolet (UV) absorption spectrophotometry (UV-1201, Shimadzu, Kyoto, Japan) following the method of Williams et al.^22^ The apparent total tract digestibility (ATTD) was calculated using the formula: Digestibility = 1 − [(concentration of nutrient in feces × concentration of Cr_2_O_3_ in the diet)/(concentration of nutrient in diet × concentration of Cr_2_O_3_ in the feces)] × 100.

### Oocyst shedding

On d 21 to 24 and 32, clean plastic was placed under each pen and fresh feces were collected. After collecting feces in a sample bag from each pen, they were stored at 4 °C until further processing following the modified procedure by Teng et al.^23^ 5 g of fecal samples were diluted with 45 mL of tap water. After proper mixing, a 1 mL mixture was taken and diluted with a 9 mL saturated salt solution. The homogenized mixture stood for 30 s to let the oocysts float. Using a water dropper pipette (Thermo Fisher Scientific, Waltham, MA), the final samples were loaded in McMaster chambers (Jorgensen Laboratories, Loveland, CO), and the total oocysts were counted. The number of oocysts per gram of feces (OPG) was calculated using the following formula: OPG count = (Number of oocysts counted/0.15) × Dilution factor. Where 0.15 is the volume of the McMaster counting chamber.

### Blood profile

Blood samples were collected from the brachial wing vein of ten broilers per treatment into a sterile syringe at d 32. The samples were collected in vacuum tubes containing K_3_EDTA for complete blood count analysis and nonheparinized tubes for serum analysis, respectively. White blood cells (WBC), heterophils, and lymphocytes were analyzed using an automatic hematology analyzer (XE2100D, Sysmex, Kobe, Japan). Immunoglobulin G (IgG) and immunoglobulin A (IgA) levels were measured in serum using an automatic biochemistry blood analyzer (Hitachi 747; Hitachi, Tokyo, Japan).

### Intestinal lesion score

On d 32, 10 broilers per treatment were euthanized, and the intestinal lesion score was measured according to the method of Dahiya et al.^24^ with slight modifications. Intestinal lesion score from middle intestine (10 cm posterior from the Meckel’s diverticulum junction; upper ileum) was scored as follows: 0 (apparently normal, no lesion), 1 (severely congested serosa and mesentery engorged with blood), 2 (thin-walled and friable intestines with small red petechiae), 3 (focal necrotic lesions), and 4 (patches of necrosis, 1 to 2 cm long).

### Fecal 16S metagenome

Fecal 16S rRNA sequencing data were analyzed using the QIIME2 next-generation microbiome bioinformatics pipeline. On d 14 and 32, the fresh feces were collected in a sample bag from each pen and stored at −20 °C until analysis. Samples were sent to Sanigen (Anyang, South Korea) for microbial sequencing using the 16S rRNA technique. All raw data was transformed in the form of QIIME2 artifacts, which contain information about the data types and sources for downstream processing. From raw sequence data, the amplicon sequence variants (ASVs) were obtained using the Divisive Amplicon Denoising Algorithm 2 (DADA2) within the QIIME2 plugin, which detects and corrects amplicon errors and filters out the potential base error and chimeric sequences.^25^ The Relative Classification Frequency Table, which represents differential abundance tests at specific taxonomic levels, was created using collapse and features within the QIIME2 plugins. The ‘diversity’ QIIME2 plugin was used to estimate alpha-diversity measurements and plots using R bioinformatics packages. This microbial diversity analysis pipeline was designed to use the ASVs table (a higher-resolution analog than the traditional operational taxonomic unit table) of the ASVs picking step as necessary input data. Analyzing the differences in species richness and evenness scores considering the sampling depth was measured using Chao1, Shannon, and Simpson. Each index estimates the V3–V4 hypervariable region of the bacterial 16S rRNA gene. A difference in relative abundance was analyzed by comparing the average bacterial proportion and composition investigated in each taxonomic ranking. Additionally, the bacterial classification accuracy according to the different amplicon regions was cross-checked by comparing the taxonomy matching rate of each ASVs taxonomy and National Center for Biotechnology Information (NCBI) bacterial reference genome database at the phylum and genus level.

### Statistical analysis

Statistical analyses and visualized graphs were performed using JMP Pro 16 (SAS Institute Inc., Cary, NC, United States) and GraphPad Prism (Version 9.1.0; GraphPad Software, San Diego, CA), respectively. All data, except fecal 16S metagenome data, were analyzed via two-way analysis of variance (ANOVA) using the Standard Least Squares model, with each pen as the experimental unit. The statistical model included the effect of the NE challenge (−C, +C), the effect of STB supplementation (0, 0.05, 0.1%), and the interaction between NE and STB. The intestinal lesion scores were determined using contingency analysis to test the relationship between categorical variables and the different combinations tested in this study. A Chi-square test was performed to determine if the different combinations had an effect on the categorial variables repartition with significance accepted at *P* < 0.05. The alpha diversity was calculated with raw counts based on Shannon estimators. For quantitative beta diversity, each treatment group was placed as the control group, and treatment groups were compared by using PROC MIXED with Dunnett’s post-hoc test. A probability level of *P* < 0.05 was indicated to be statistically significant.

## Result

### Growth performance

On d 14, the supplementation of 0.05% STB significantly increased (*P* < 0.05) BW compared to the supplementation of 0% STB (Table 2). On d 7 to 14, the supplementation of 0.05% STB resulted in significantly higher (*P* < 0.05) BWG and lower (*P* < 0.05) FCR compared to the supplementation of 0 and 0.1% STB. On d 21 and 32 (d 7 and 18 post-inoculation [PI]), the NE challenge significantly decreased (*P* < 0.05) BW compared to the non-challenge group. On d 32, the supplementation of 0.05 and 0.10% STB significantly increased (*P* < 0.05) BW compared to the non-supplementation group. On d 14 to 21 (d 0 to 7 PI), the NE challenge significantly reduced (*P* < 0.05) BWG and increased (*P* < 0.05) FCR compared to the non-challenge group. On d 14 to 21 and d 21 to 32 (d 0 to 7 PI and d 7 to 18 PI), the supplementation of 0.05% STB significantly decreased (*P* < 0.05) FCR compared to the non-supplementation group. During the entire period, the NE challenge significantly reduced (*P* < 0.05) BWG and increased (*P* < 0.05) FCR compared to the non-challenge group. The supplementation of STB significantly increased (*P* < 0.05) BWG compared to the non-supplementation group. Also, the supplementation of 0.05% STB significantly decreased (*P* < 0.05) FCR compared to the supplementation of 0 and 0.1% STB. There were no interactions between the NE challenge and the supplementation of STB in growth performance.

**Table 2.** Effects of stimbiotic (STB) on growth performance of broilers challenged with necrotic enteritis (NE).

Items	−C			+C			SEM	C		STB			*P*-value		
	0	0.05	0.10	0	0.05	0.10		−	+	0	0.05	0.10	C	STB	C × STB
**Pre**															
BW, g															
d 0	34.67	34.67	34.67	35.13	35.07	34.67	0.287	34.67	34.96	34.90	34.87	34.67		0.689	
d 7	150.67	158.67	161.67	160.67	160.83	162.00	4.566	157.00	161.17	155.67	159.75	161.83		0.383	
d 14	379.33	410.67	391.67	382.00	412.50	392.33	8.354	393.89	395.61	380.67b	411.58a	392.00ab		0.002	
d 0 to 7															
BWG, g	121.52	123.95	126.87	125.46	126.07	127.18	4.796	124.11	126.23	123.49	125.01	127.02		0.754	
FI, g	141.69	143.77	150.86	142.54	144.74	136.63	3.729	145.44	141.30	142.11	144.25	143.74		0.836	
FCR	1.17	1.16	1.19	1.14	1.15	1.07	0.045	1.17	1.12	1.15	1.15	1.13		0.893	
d 7 to 14															
BWG, g	223.00	252.00	230.00	221.33	251.67	230.33	7.659	235.00	234.44	222.17b	251.83a	230.17b		0.001	
FI, g	319.33	318.96	327.11	309.09	330.00	321.24	5.261	321.80	320.11	314.21	324.48	324.17		0.091	
FCR	1.43	1.27	1.42	1.40	1.31	1.39	0.047	1.37	1.37	1.41a	1.29b	1.41a		0.045	
**Post**															
BW, g															
d 21	869.33	901.33	890.33	827.33	864.58	844.67	12.044	887.00	845.53	848.33b	882.96a	867.50ab	<0.001	0.023	0.935
d 32	1662.33	1741.67	1726.67	1607.67	1691.25	1646.67	21.798	1710.22	1648.53	1635.00b	1716.46a	1686.67a	0.001	0.002	0.763
d 14 to 21															
BWG, g	490.00	490.67	498.67	445.33	450.42	452.33	9.090	493.11	449.36	467.67	470.54	475.50	<0.001	0.676	0.944
FI, g	734.33	730.33	745.67	741.33	711.25	718.67	11.540	736.78	723.75	737.83	720.79	732.17	0.173	0.342	0.301
FCR	1.50	1.49	1.50	1.66	1.58	1.59	0.016	1.49	1.61	1.58a	1.53b	1.54ab	<0.001	0.011	0.052
d 21 to 32															
BWG, g	793.00	840.33	836.33	780.33	826.67	802.00	15.449	823.22	803.00	786.67b	833.50a	819.17ab	0.115	0.012	0.727
FI, g	1316.15	1308.41	1322.62	1283.84	1313.12	1307.19	24.169	1315.73	1301.38	1300.00	1310.76	1314.90	0.471	0.811	0.752
FCR	1.66	1.56	1.58	1.65	1.59	1.63	0.026	1.60	1.62	1.65a	1.57b	1.61ab	0.315	0.011	0.298
d 0 to 32															
BWG, g	1627.52	1706.95	1691.87	1572.46	1656.41	1611.84	21.632	1675.45	1613.57	1599.99b	1681.68a	1651.86a	0.001	0.002	0.763
FI, g	2511.50	2501.47	2546.25	2476.81	2499.10	2483.72	32.447	2519.74	2486.54	2494.15	2500.28	2514.99	0.216	0.799	0.659
FCR	1.54	1.47	1.50	1.58	1.51	1.54	0.012	1.50	1.54	1.56a	1.49c	1.52b	<0.001	<0.001	0.881

−C: non-challenge; +C: challenge with NE; 0: basal diet; 0.05: basal diet with 0.05% STB; 0.10: basal diet with 0.10% STB; BW: body weight; BWG: body weight gain; FI: feed intake; FCR: feed conversion ratio; SEM: standard error of means.

a–c Means with different letters are significantly differ (*P* < 0.05).

### Nutrient digestibility

On d 14, the supplementation of 0.05% STB significantly increased (*P* < 0.05) DM and GE digestibility (Table 3). On d 32 (d 18 PI), the NE challenge significantly decreased (*P* < 0.05) DM, CP, and GE digestibility compared to the non-challenge group. The supplementation of 0.05% STB significantly increased (*P* < 0.05) GE digestibility compared to the non-supplementation group. There was an interaction between the NE challenge and the supplementation of STB in GE digestibility on d 32.

**Table 3.** Effects of stimbiotic (STB) on nutrient digestibility of broilers challenged with necrotic enteritis (NE).

Items, %	−C			+C			SEM	C		STB			*P*-value		
	0	0.05	0.10	0	0.05	0.10		−	+	0	0.05	0.10	C	STB	C × STB
**D 14**															
DM	66.30	66.94	65.98	65.02	67.00	66.56	0.484	66.41	66.19	65.66b	66.97a	66.27ab		0.032	
CP	70.84	70.52	70.98	70.64	70.40	70.84	1.145	70.78	70.63	70.74	70.46	70.91		0.924	
GE	68.22	69.78	69.06	68.38	70.44	68.86	0.600	69.02	69.23	68.30b	70.11a	68.96ab		0.014	
**D 32**															
DM	68.84	70.02	68.36	65.40	67.18	67.06	0.766	69.07	66.55	67.12	68.60	67.71	<0.001	0.160	0.361
CP	74.24	75.06	72.42	69.22	71.68	71.26	1.006	73.91	70.72	71.73	73.37	71.84	<0.001	0.199	0.166
GE	71.78ab	73.06a	70.20bc	65.50d	69.10c	68.14 cd	0.637	71.68	67.58	68.64a	71.08a	69.17b	<0.001	<0.001	0.007

−C: non-challenge; +C: challenge with NE; 0: basal diet; 0.05: basal diet with 0.05% STB; 0.10: basal diet with 0.10% STB; DM: dry matter; CP: crude protein; GE: gross energy; SEM, standard error of means.

a–d Means with different letters are significantly differ (*P* < 0.05).

### Oocyst shedding

There were no oocysts detected in the feces obtained from the non-challenge group (Table 4). The pattern of oocyst shedding showed a decrement during d 21 to 24 and d 32 (d 7 to 10 PI and d 18 PI) in all challenge groups. The supplementation of STB significantly decreased (*P* < 0.05) OPG count compared to the non-supplementation group on d 21, 22, and 32 (d 7, 8, and 18 PI). On d 32 (d 18 PI), the supplementation of 0.05% STB significantly decreased (*P* < 0.05) OPG count compared to the supplementation of 0 and 0.1% STB. There was an interaction between the NE challenge and the supplementation of STB in OPG count on d 21, 22, 23, and d 32. The supplementation of STB with NE challenge significantly decreased (*P* < 0.05) OPG count compared to the non-supplementation group with NE challenge. Also, the supplementation of 0.05% STB with NE challenge significantly decreased (*P* < 0.05) OPG count compared to the supplementation of 0 and 0.1% STB with NE challenge.

**Table 4.** Effects of stimbiotic (STB) on oocysts per gram in feces (OPG) in broilers challenged with necrotic enteritis (NE).

Items, ×10^3^	−C			+C			SEM	C		STB			*P*-value		
	0	0.05	0.10	0	0.05	0.10		−	+	0	0.05	0.10	C	STB	C × STB
OPG count															
d 21	0.00c	0.00c	0.00c	345.60a	236.00b	254.47b	18.520	0.00	279.02	172.80a	118.00b	127.73b	<0.001	0.010	0.010
d 22	0.00c	0.00c	0.00c	283.20a	164.00b	176.53b	7.666	0.00	207.91	141.60a	82.00b	88.27b	<0.001	<0.001	<0.001
d 23	0.00c	0.00c	0.00c	180.27a	140.27b	161.60ab	5.918	0.00	160.71	90.13a	70.13b	80.80ab	<0.001	0.006	0.006
d 24	0.00	0.00	0.00	138.13	117.87	125.33	5.207	0.00	127.11	69.07	58.93	62.67	<0.001	0.154	0.154
d 32	0.00d	0.00d	0.00d	103.20a	64.80c	83.73b	2.838	0.00	83.91	51.60a	32.40c	41.87b	<0.001	<0.001	<0.001

−C: non-challenge; +C: challenge with NE; 0: basal diet; 0.05: basal diet with 0.05% STB; 0.10: basal diet with 0.10% STB; SEM: standard error of means.

a–d Means with different letters are significantly differ (*P* < 0.05).

### Blood profile

On d 32 (d 18 PI), the NE challenge significantly increased (*P* < 0.05) heterophil levels compared to the non-challenge group (Table 5). The supplementation of 0.05% STB significantly decreased (*P* < 0.05) heterophil levels compared to the non-supplementation group. There were no interactions between the NE challenge and the supplementation of STB in blood profiles.

**Table 5.** Effects of stimbiotic (STB) on blood profile of broilers challenged with necrotic enteritis (NE).

Items	−C			+C			SEM	C		STB			*P-*value		
	0	0.05	0.10	0	0.05	0.10		−	+	0	0.05	0.10	C	STB	C × STB
WBC, 10³/ μl	25.70	26.42	27.59	26.11	27.20	25.82	1.547	26.57	26.38	25.91	26.81	26.71	0.880	0.816	0.675
Heterophil, %	22.70	19.66	21.52	27.38	24.34	23.12	1.174	21.29	24.95	25.04a	22.00b	22.32ab	<0.001	0.023	0.325
Lymphocyte, %	58.66	59.90	58.98	55.66	56.74	56.74	1.817	59.18	56.38	57.16	58.32	57.86	0.065	0.814	0.964
IgG, mg/dL	1.20	1.00	1.40	1.20	1.20	1.20	0.128	1.20	1.20	1.20	1.10	1.30	1.000	0.301	0.301
IgA, mg/dL	1.60	2.00	1.40	1.40	1.40	1.20	0.255	1.67	1.33	1.50	1.70	1.30	0.116	0.301	0.666

−C: non-challenge; +C: challenge with NE; 0: basal diet; 0.05: basal diet with 0.05% STB; 0.10: basal diet with 0.10% STB; WBC: white blood cell; IgG: immunoglobulin G; IgA: immunoglobulin A; SEM: standard error of means.

a,b Means with different letters are significantly differ (*P* < 0.05).

### Intestinal lesion score

**Figure 1.**
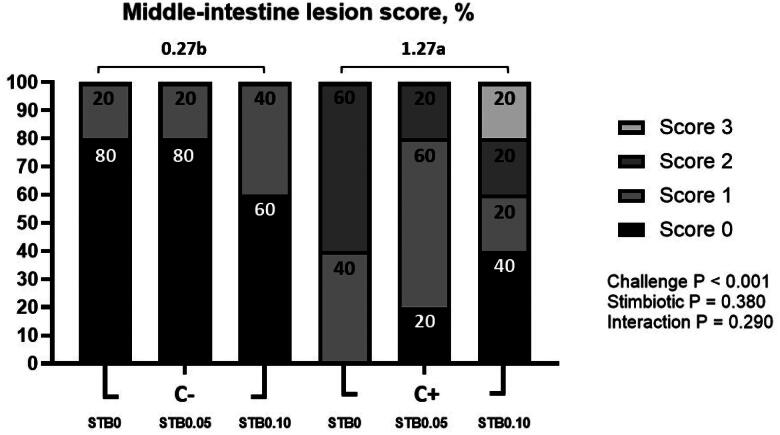
Effects of stimbiotic (STB) on intestine lesion score of broilers challenged with necrotic enteritis (NE). −C: non-challenge; +C: challenge with NE; 0: basal diet; 0.05: basal diet with 0.05% STB; 0.10: basal diet with 0.10% STB. *n* = 10 broilers/treatment. *X*^2^ = 41.829, *P* < 0.001. Numbers inside the bar indicates percentage of score out of total (100%). ^a,b^Mean scores followed by different superscripts in the bar graph indicate statistically significance by student’s t-test (*P* < 0.05).

On d 32 (d 18 PI), the NE challenge significantly increased (*P* < 0.05) the middle-intestine lesion score compared to the non-challenge group (Fig. 1). There were no interactions between the NE challenge and the supplementation of STB in intestinal lesion score.

### Diversity of the fecal microbiome

On d 14, the supplementation of 0.05% STB was significantly higher (*P* < 0.05) in alpha-diversity parameters (Shannon and Simpson) than the supplementation of 0 and 0.1% STB (Table 6). On d 32 (d 18 PI), the NE challenge had significantly lower (*P* < 0.05) Chao1 indices than the non-challenge group. The supplementation of 0.05% STB was significantly higher (*P* < 0.05) in Chao1 indices than the supplementation of 0 and 0.1% STB. There was an interaction between the NE challenge and the supplementation of STB in alpha-diversity on d 32. The supplementation of 0.05% STB with NE challenge was significantly higher (*P* < 0.05) in Chao1 and Shannon indices than the supplementation of 0.1% STB with NE challenge.

**Table 6.** Effects of stimbiotic (STB) on alpha-diversity of broilers challenged with necrotic enteritis (NE).

Items	−C			+C			SEM	C		STB			*P-*value		
	0	0.05	0.10	0	0.05	0.10		−	+	0	0.05	0.10	C	STB	C × STB
D 14															
Chao1	235.00	297.91	206.77	380.39	319.90	318.52	3.158	246.56	339.60	307.70a	308.90a	262.64b		<.0001	
Shannon	3.38	5.90	3.84	6.17	5.83	6.09	0.045	4.37	6.03	4.78c	5.86a	4.97b		<.0001	
Simpson	0.83	0.96	0.87	0.96	0.95	0.97	0.003	0.89	0.96	0.90c	0.95a	0.92b		<.0001	
D 32															
Chao1	195.42d	410.96b	476.42a	439.62ab	348.47c	205.25d	8.891	360.93	331.11	317.52b	379.72a	340.83b	0.001	<0.001	<0.001
Shannon	3.23d	5.55bc	6.79a	6.38ab	5.10c	3.05d	0.201	5.19	4.84	4.80	5.32	4.92	0.057	0.056	<0.001
Simpson	0.81ab	0.92a	0.98a	0.98a	0.92a	0.65b	0.042	0.91	0.85	0.89	0.92	0.82	0.115	0.078	<0.001

−C: non-challenge; +C: challenge with NE; 0: basal diet; 0.05: basal diet with 0.05% STB; 0.10: basal diet with 0.10% STB; SEM: standard error of means.

a–d Means with different letters are significantly differ (*P* < 0.05).

**Figure 2.**
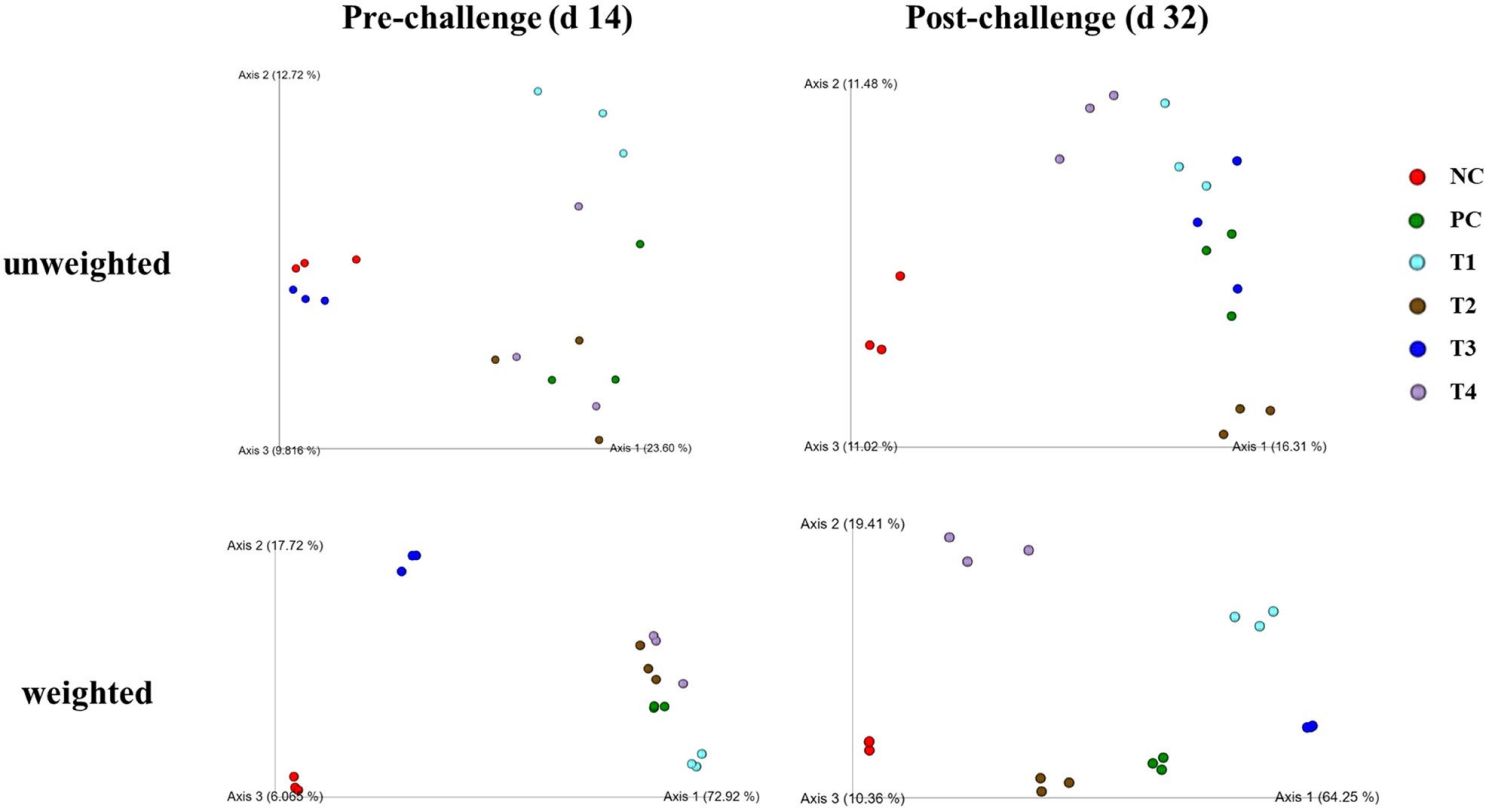
Visualized beta-diversity indices including unweighted and weighted emperor in negative control (NC): basal diet; positive control (PC): basal diet with necrosis enteritis (NE) challenge; T1: NC + 0.05% STB; T2: PC + 0.05% STB; T3: NC + 0.10% STB; T4: PC + 0.10% STB.

**Figure 3.**
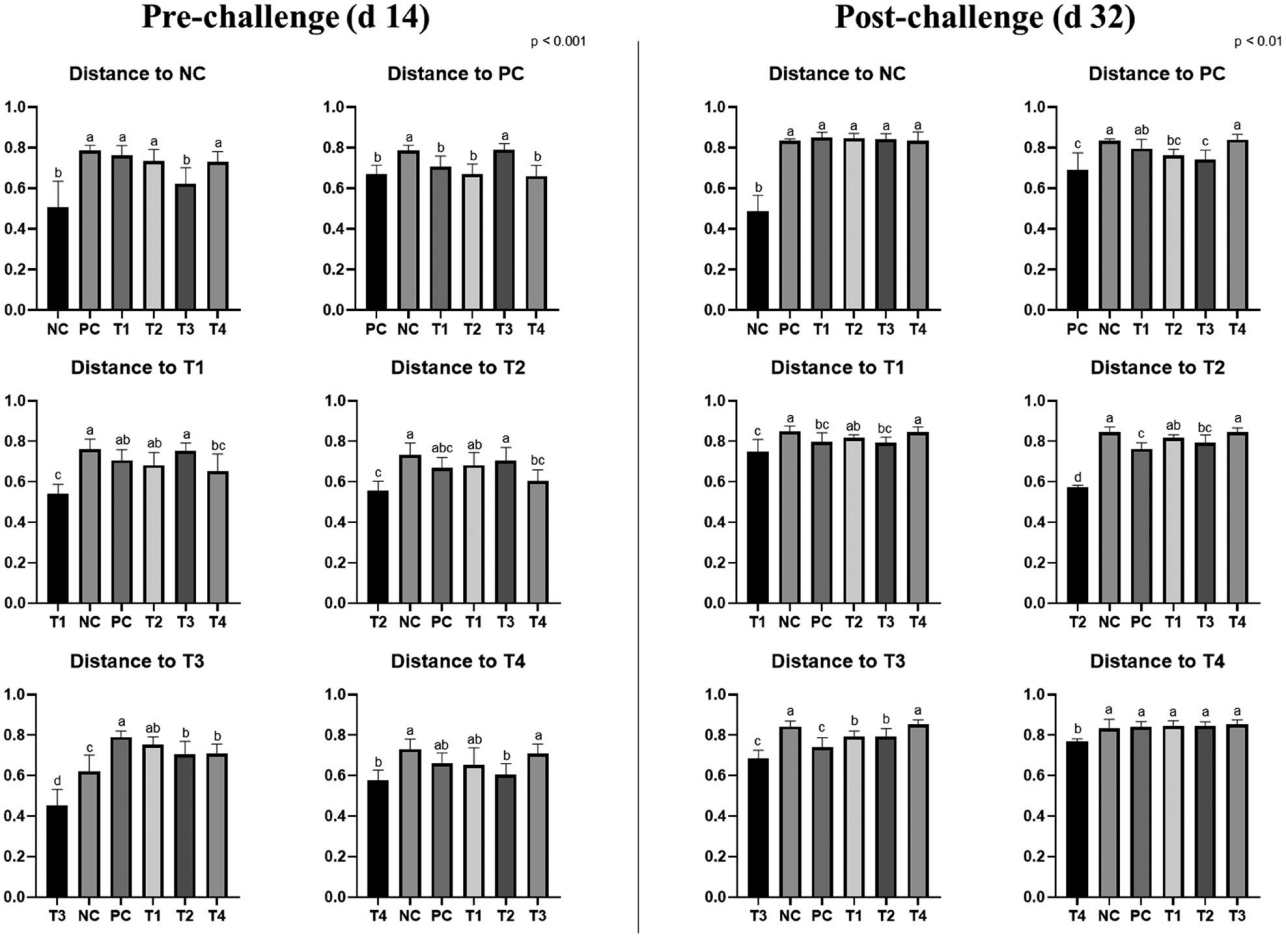
Unweighted Unifrac measurement in negative control (NC): basal diet; positive control (PC): basal diet with necrosis enteritis (NE) challenge; T1: NC + 0.05% STB; T2: PC + 0.05% STB; T3: NC + 0.10% STB; T4: PC + 0.10% STB. Each treatment group was placed as the control group, and treatment groups were compared by using one-way PROC MIXED with Dunnett’s post-hoc test.

**Figure 4.**
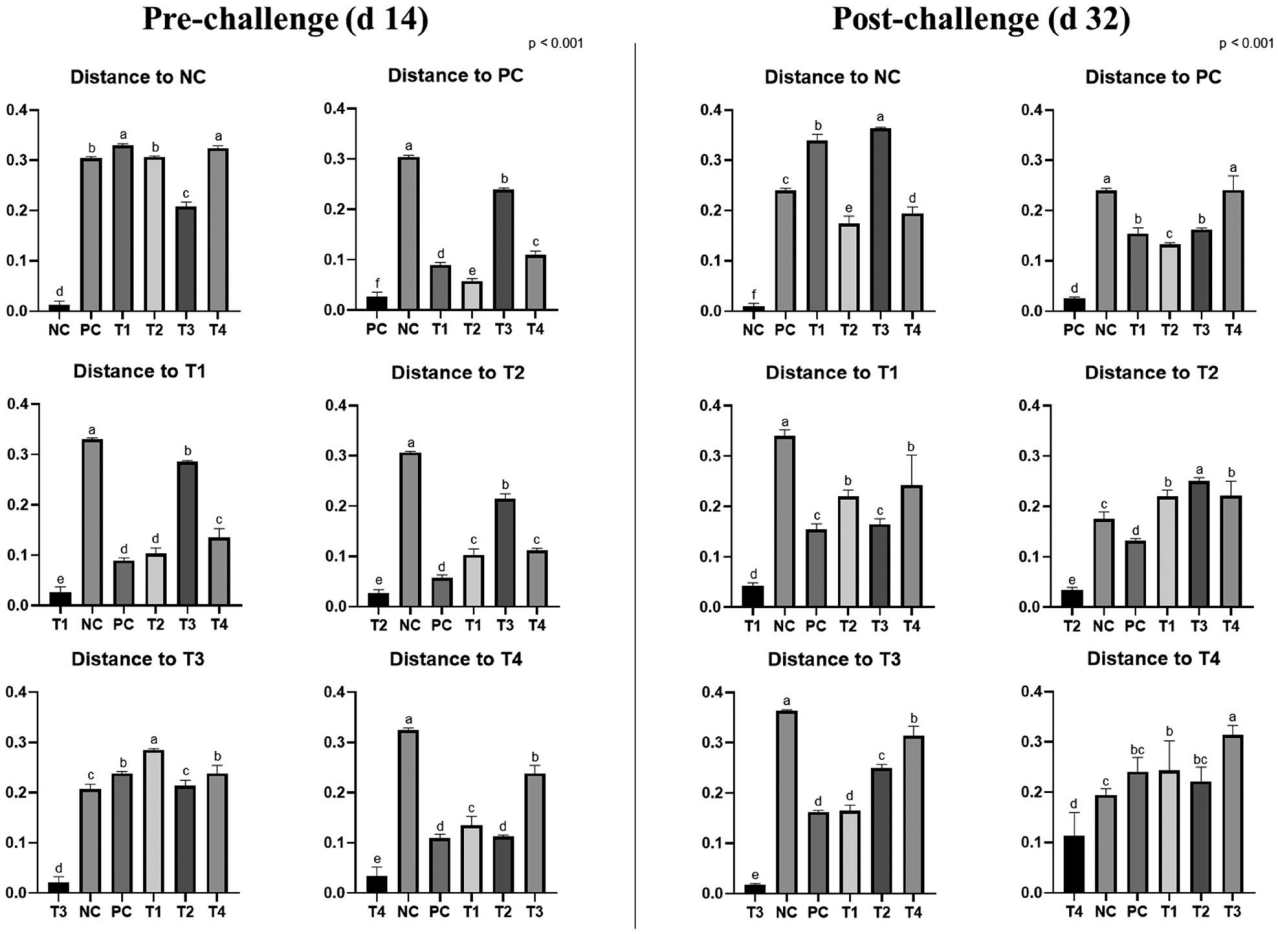
Weighted unifrac measurement in negative control (NC): basal diet; positive control (PC): basal diet with necrosis enteritis (NE) challenge; T1: NC + 0.05% STB; T2: PC + 0.05% STB; T3: NC + 0.10% STB; T4: PC + 0.10% STB. Each treatment group was placed as the control group, and treatment groups were compared by using one-way PROC MIXED with Dunnett’s post-hoc test.

On d 14, the supplementation of 0.1% STB showed a difference (*P* < 0.05) from other groups in unweighted and weighted unifrac distance (Figs. 2–4). Also, the non-supplementation group showed a difference (*P* < 0.05) from the STB-supplementation group in weighted unifrac distance. On d 32, the NE challenge group differed from the non-challenge group in unweighted and weighted unifrac distances. The supplementation of 0.05% STB with NE challenge showed a difference (*P* < 0.05) from the other groups in unweighted and weighted unifrac distance.

### Relative abundance

At the phylum level, the supplementation of STB significantly decreased (*P* < 0.05) the abundance of *Bacteroidota* and *Proteobacteria* compared to the non-supplementation group on d 14 (Table 7). On d 32, the NE challenge significantly increased (*P* < 0.05) the abundance of *Proteobacteria* compared to the non-challenge group. The supplementation of 0.1% STB significantly decreased (*P* < 0.05) the abundance of *Firmicutes* and *Bacteroidota* compared to the supplementation of 0.05% STB. There was an interaction between the NE challenge and the supplementation of STB in the abundance of *Actinobacteriota*, *Firmicutes*, *Bacteroidota*, *Proteobacteria*, *Bacteria*, and *Cyanobacteria* on d 32.

**Table 7.** Effects of stimbiotic (STB) on relative abundance of fecal microbiota at the phylum level of broilers challenged with necrotic enteritis (NE).

Items, %	−C			+C			SE	C		STB			*P*-value		
	0	0.05	0.10	0	0.05	0.10		−	+	0	0.05	0.10	C	STB	C × STB
**D 14**															
*Actinobacteriota*	0.12	0.07	0.03	0.07	0.31	0.08	0.007	0.15	0.07	0.09b	0.05c	0.20a		<0.001	
*Firmicutes*	62.60	97.73	99.02	97.26	90.00	98.11	0.396	83.87	97.70	80.17c	98.14a	94.06b		<0.001	
*Bacteroidota*	0.04	0.04	0.02	0.00	0.00	0.01	0.004	0.02	0.02	0.04a	0.01b	0.01b		<0.001	
*Proteobacteria*	36.49	2.00	0.80	2.55	9.68	1.70	0.406	15.66	2.09	19.25a	1.68c	5.69b		<0.001	
*Bacteria*	0.01	0.01	0.02	0.01	0.00	0.01	0.002	0.01	0.01	0.01a	0.01a	0.004b		<0.001	
*Cyanobacteria*	0.58	NS	NS	NS	NS	NS	0.014	0.19	NS	0.29a	NS	NS		<0.001	
*Verrucomicrobiota*	0.15	0.01	NS	0.00	0.00	NS	0.007	0.05	0.00	0.08a	0.001b	0.0001b		<0.001	
Rest	0.01	0.14	0.11	0.11	0.01	0.10	0.012	0.04	0.12	0.08	0.11	0.05		0.002	
**D 32**															
*Actinobacteriota*	0.07	1.27	0.40	0.12	0.57	0.02	0.037	0.35	0.47	0.67a	0.26b	0.29b	0.002	<0.001	<0.001
*Firmicutes*	60.24	89.34	69.18	81.42	96.92	28.82	2.600	75.45	66.53	74.79a	75.30a	62.87b	0.001	<0.001	<0.001
*Bacteroidota*	0.01	2.22	26.02	4.43	0.79	11.72	2.657	8.94	6.12	1.11b	15.23a	6.25b	0.218	<0.001	<0.001
*Proteobacteria*	39.16	6.38	2.30	12.98	0.41	59.24	4.931	13.96	26.20	22.77a	7.64b	29.83a	0.010	0.002	<0.001
*Bacteria*	0.01	0.03	0.22	0.29	0.18	0.04	0.032	0.14	0.12	0.02c	0.25a	0.11b	0.514	<0.001	0.015
*Cyanobacteria*	0.40	0.59	1.37	0.18	0.81	0.03	0.057	0.86	0.27	0.49b	0.78a	0.42b	<0.001	<0.001	<0.001
*Verrucomicrobiota*	0.10	0.02	0.29	0.29	0.16	0.00	0.031	0.18	0.11	0.06b	0.29a	0.08b	0.010	<0.001	0.076
Rest	0.02	0.16	0.22	0.29	0.17	0.13	0.019	0.13	0.19	0.09c	0.25a	0.15b	0.002	<0.001	0.002

−C: non-challenge; +C: challenge with NE; 0: basal diet; 0.05: basal diet with 0.05% STB; 0.10: basal diet with 0.10% STB; SEM: standard error of means.

a–c Means with different letters are significantly differ (*P* < 0.05).

**Figure 5.**
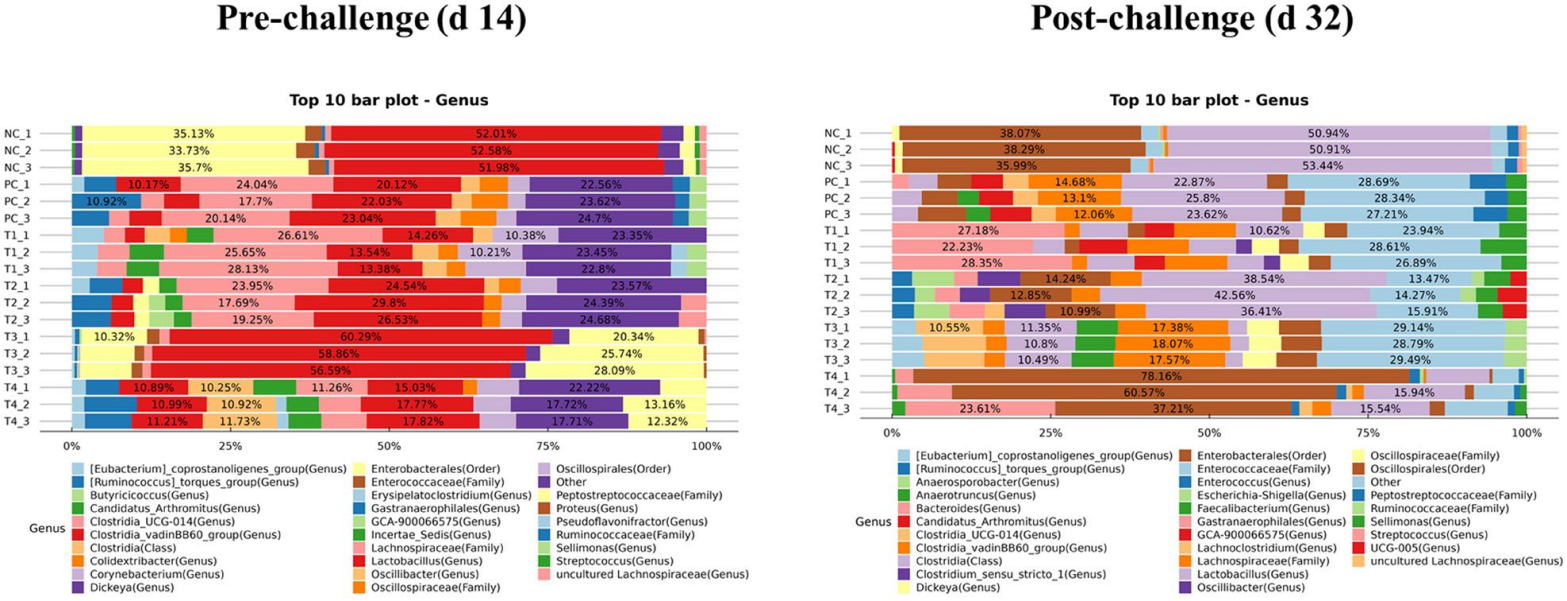
16S rRNA gene analysis revealed the relative abundance of fecal bacterial community at the genus level in negative control (NC): basal diet; positive control (PC): basal diet with necrosis enteritis (NE) challenge; T1: NC + 0.05% STB; T2: PC + 0.05% STB; T3: NC + 0.10% STB; T4: PC + 0.10% STB.

At the genus level, the supplementation of 0.05% STB significantly increased (*P* < 0.05) the abundance of *Lachnospiraceae* and decreased (*P* < 0.05) the abundance of *Enterobacterales* compared to the non-supplementation group on d 14 (Table 8; Fig. 5). On d 32, the NE challenge significantly decreased (*P* < 0.05) the abundance of *Lachnospiraceae* compared to the non-challenge group. The supplementation of 0.05% STB significantly decreased (*P* < 0.05) the abundance of *Enterobacterales* compared to the supplementation of 0 and 0.1% STB. There was an interaction between the NE challenge and the supplementation of STB in the relative abundance of fecal microbiota on d 32.

**Table 8.** Effects of stimbiotic (STB) on relative abundance of fecal microbiota at the genus level of broilers challenged with necrotic enteritis (NE).

Items, %	−C			+C			SE	C		STB			*P*-value		
	0	0.05	0.10	0	0.05	0.10		−	+	0	0.05	0.10	C	STB	C × STB
**D 14**															
*Lactobacillus*	52.19	21.73	13.73	26.96	58.58	16.87	0.931	41.50	21.85	36.96a	20.34b	37.73a		<0.001	
*Lachnospiraceae*	0.88	20.63	26.80	20.30	1.42	8.32	1.268	9.70	16.42	10.75b	23.55a	4.87c		<0.001	
*Enterobacterales*	34.85	NS	NS	2.46	9.02	NS	0.362	14.62	0.82	17.43a	1.23c	4.51b		<0.001	
*Ruminococcus_torques_group*	NS	7.27	NS	5.84	0.50	6.97	0.848	0.17	6.70	3.64	2.92	3.73		0.592	
*Sellimonas*	NS	2.74	2.06	NS	NS	NS	0.420	0.69	0.91	1.37a	1.03ab	NS		0.018	
*Peptostreptococcaceae*	2.05	NS	NS	NS	24.72	10.94	1.197	8.92	3.65	1.02b	NS	17.83a		<0.001	
*Oscillospirales*	NS	3.10	10.05	4.37	NS	6.07	0.329	3.35	4.51	1.55c	7.21a	3.03b		<0.001	
*Eubacterium_coprostanoligenes*	NS	0.66	4.39	0.94	0.46	2.07	0.487	1.61	1.22	0.33b	2.66a	1.27b		0.002	
Rest	10.03	43.87	42.99	39.14	5.31	48.76	1.199	19.44	43.93	26.95b	41.07a	27.04b		<0.001	
**D 32**															
*Lactobacillus*	51.77	24.10	7.93	39.17	2.90	13.80	1.326	20.86	25.69	37.93a	23.55b	8.35c	<0.001	<0.001	<0.001
*Lachnospiraceae*	0.55	13.28	9.77	4.71	17.67	1.75	0.440	9.33	6.58	6.92b	7.24b	9.71a	<0.001	<0.001	<0.001
*Enterobacterales*	37.45	6.19	1.64	12.70	NS	58.65	4.887	13.03	25.85	21.82a	7.17b	29.32a	0.008	0.002	<0.001
*Ruminococcus_torques_group*	NS	NS	NS	3.41	NS	NS	0.054	NS	1.14	NS	1.71a	NS	<0.001	<0.001	<0.001
*Sellimonas*	NS	3.08	5.22	3.80	NS	0.94	0.488	1.74	2.61	1.54b	4.51a	0.47b	0.051	<0.001	0.002
*Peptostreptococcaceae*	1.80	4.92	NS	NS	NS	1.01	0.268	0.60	1.98	3.36a	NS	0.51b	<0.001	<0.001	<0.001
*Oscillospirales*	NS	3.11	3.38	NS	6.46	1.43	0.235	3.28	1.51	1.56b	1.69b	3.95a	<0.001	<0.001	<0.001
*Bacteroides*	NS	0.90	25.92	4.41	NS	11.70	2.675	8.64	5.67	0.45b	15.16a	5.85b	0.199	<0.001	<0.001
Rest	8.43	44.43	46.15	31.80	72.97	10.72	1.537	45.52	28.98	26.43b	38.97a	41.84a	<0.001	<0.001	<0.001

−C: non-challenge; +C: challenge with NE; 0: basal diet; 0.05: basal diet with 0.05% STB; 0.10: basal diet with 0.10% STB; SEM: standard error of means.

a–c Means with different letters are significantly differ (*P* < 0.05).

## Discussion

The prohibition of antimicrobial use in poultry diets has led to the resurgence of NE, resulting in elevated mortality rates and economic losses.^26^ Following the prohibition of incorporating growth-promoting antibiotics into animal diets in the European Union, the poultry industry has faced economically significant challenges due to *C. perfringens*-induced necrotic enteritis and associated subclinical diseases.^9^^,^^27^ In this experiment, the NE challenge group exhibited a significant decrease in BW compared to the non-challenge group. Throughout the entire period, the NE challenge significantly reduced BWG compared to the non-challenge group. The decrease in growth performance indicates that the NE challenge was well-induced in this study. Also, in a previous study, induction of NE in response to *C. perfringens* infection resulted in decreased growth performance, including final BW, BWG, and FI, and higher diarrhea and intestinal lesion scores compared to control broilers.^28^ In this study, the NE challenge group exhibited a significant increase in the middle-intestine lesion score compared to the non-challenged group. Intestinal lesions caused by coccidiosis, along with NE resulting from *C. perfringens*, continue to pose a major problem.^27^ Transmission of coccidia occurs through the fecal-oral route, involving the shedding of spore-free oocysts from the intestinal mucosa, which are then excreted in the feces.^29^ For excreted oocysts to become infective, they must undergo sporulation, a process requiring oxygen, moisture, and warmth.^30^ In this study, middle-intestine lesion scores were induced due to the NE challenge, and oocyst shedding was confirmed in the stool due to the inflammatory reaction. Previous research has shown that oocysts can induce gut damage, resulting in increased nutrient release into the lumen, which significantly promotes the growth and proliferation of *C. perfringens*.^2^ The NE triggers robust local inflammatory reactions, evident at the interface between the basal domain of enterocytes and the hyperemic lamina propria.^31^ Heterophils play a crucial role as the primary effector cells in the avian host defense against bacterial, viral, fungal, and parasitic infections, highlighting their significance.^32^ In this study, a significant increase in heterophils following the NE challenge was confirmed. Hence, the induction of NE proved successful through the growth performance, digestibility, inflammatory response, and middle-intestine lesions in broilers.

The objective of this study was to investigate the STB as a method to prevent NE-induced decline in growth performance and immunity. Supplementing with STB resulted in a significant increase in BWG compared to the non-supplemented group. Additionally, the inclusion of 0.05% STB significantly reduced FCR. Prior research has similarly demonstrated that the inclusion of STB in a broiler diet enhances growth performance and mitigates the inflammatory response induced by NE.^19^ However, in this experiment, there was no observed interaction between NE challenge and supplementation with STB regarding growth performance. An additional study suggests that supplementing with STB could enhance growth performance, intestinal development, and barrier function in broilers.^33^ In this study, the addition of 0.05% STB significantly increased GE digestibility compared to non-addition, revealing an interaction between NE challenge and STB supplementation in GE digestibility. These results demonstrate the effectiveness of STB supplementation in improving growth performance and nutrient digestibility in broilers. The improvement in digestibility and growth is attributed to the enhanced availability of NSP facilitated by STB. The NSP is a substantial part of poultry diets and is not efficiently digested by the body’s internal enzyme systems.^34^ In a prior study, it was noted that including NSP in the diet led to elevated intestinal viscosity in broilers, causing a decrease in interaction between enzymes and substrates.^35^^,^^36^ Arabinoxylan, one of the major NSP, generates XOS when decomposed, which can improve nutrient digestibility and have a positive effect on the intestinal microflora.^37^ The XOS are inclined to function as activators of specific bacteria in the gastrointestinal tract rather than serving directly as a quantitative prebiotic.^38^ This mechanism can potentially enhance the production of short-chain fatty acids, including butyrate, contributing to gut health.^39^^,^^40^ In this study, the supplementation of STB partially mitigated the decline in growth performance and alleviated the induced inflammatory response caused by the NE challenge. As a result of this experiment, intestinal lesions induced by NE challenge and oocyst shedding due to inflammatory response were significantly reduced by STB supplementation. Furthermore, the supplementation of 0.05% STB in the diet led to a significant reduction in heterophils compared to the non-supplemented group. This suggests that STB supplementation alleviates the inflammatory response. Also, in this study. when STB was added in broilers, the diversity of the fecal microbiome community increased depending on the addition level, the non-supplementation group showed a difference from the STB-supplementation group in beta-diversity. At the genus level, an interaction was noted between the NE challenge and STB supplementation in the relative abundance of fecal microbiota. This indicates that the supplementation of STB influences the balance and composition of intestinal microbiota, highlighting the prebiotic performance of XOS. The intestinal administration of XOS can enhance intestinal morphology, structure, and the population of intestinal microbes.^41^ The XOS supplementation may offer a receptor on the intestinal surface for gram-negative pathogens.^42^ This could serve as an attachment site for Gram-negative pathogens promoted by *C. perfringens*, preventing bacterial attachment to the enterocytes.^43^

As a result, supplementing STB to broiler diets can improve broiler growth performance and increase nutrient digestibility. It also inhibits inflammation by reducing oocyst shedding excretion and lowers inflammation levels. Additionally, we confirmed its potential to suppress harmful intestinal bacteria and increase intestinal microbial diversity. However, the correlation between STB supplementation and NE induction and inhibition has not been demonstrated, and the exact mechanism of improvement in intestinal microbial diversity and intestinal health has not been identified. Therefore, further studies are needed to clarify this correlation.

## Conclusion

The NE had a negative impact on broilers by reducing broiler growth performance and energy digestibility, increasing intestinal lesion scores, fecal oocyst count, and heterophil levels in the blood. Supplementation of STB was found to positively regulate the intestinal microflora, reduce the number of oocysts in the feces, and improve growth performance (reduce FCR). In conclusion, it is thought that supplementing 0.05% STB in a diet can reduce the economic loss caused by NE in the broiler industry by alleviating the negative effects of NE and positively controlling fecal microbiota.
